# Supplementary material for: Predictive variables of prescription opioid misuse in patients with chronic noncancer pain. Development of a risk detection scale: A registered report protocol
Source: PLoS One. 2021 May 13;16(5):e0251586. doi: 10.1371/journal.pone.0251586 (PMC8118327; doi:10.1371/journal.pone.0251586)
Supplement: Attachment — Submitted filename: RESPONSE TO REVIEWERS_V3.docx [file pone.0251586.s001.docx]

**Málaga, March 2021**

***PLOS ONE***

Dear Dr. Vijayaprakash Suppiah,

We very much appreciate your new suggestions regarding our paper (***PONE-D-21-02639
Predictive variables of prescription opioid misuse in patients with chronic noncancer pain. Development of a risk detection scale: A registered report protocol).*** We have revised the paper accordingly and hope the new version is now acceptable for publication.

This is a Registered Report Protocol and so we do not yet have data available to share (not even a minimal data set). However, I confirm that we will be able to make our data available when our study is completed and if it is accepted for publication.

In the following, we explain how the reviewers' suggestions have been taken into account in the new version. Changes in the text can be found in red.

**Journal’s requirements:**

*Please ensure that you include a title page* *within your main document. You should list all authors and all affiliations as per our author instructions and clearly indicate the corresponding author*

**Answer.** The title page has been included within our main document

*Please include your tables as part of your main manuscript and remove the individual files. Please note that supplementary tables (should remain/ be uploaded) as separate "supporting information" files.*

**Answer.** Following the Journal´s requirements, the tables have been included as part of our main manuscript.

**Reviewer comments:**

**Reviewer #1 (R#1)**

*This referee think that this study protocol is well designed and the manuscript is well written, but there are several grammatical errors on the manuscript.*

Thank you very much for your positive comments. A professional translator (native English speaker) has reviewed again the text.

**Reviewer #2 (R#2)**

*1. The first major concern that should be addressed is about the concept of "misuse" defined in the title. Opioids in chronic pain patients can lead not only to misuse but also to abuse. Please discuss better this two problems and define better how authors' protocols can help clinicians to identify the two different problems*

**Answer**. We agree with reviewer. In fact, we just wanted to assess the overuse of opioids prescribed. New text has been included in the introduction section (new text shown in red), explaining that the misuse of opioids "... has been defined as the use of opioids in a manner other than how they are prescribed (Martel, Edwards, & Jamison, 2020). Prescription opioid misuse may involve a wide range of behaviours, including overuse (Martel et al., 2020). In our project, misuse is defined as the overuse of prescribed opioids (i.e., taking more opioids than prescribed)” (lines 79-82)

New reference:

14- Martel MO, Edwards RR, Jamison RN. The relative contribution of pain and psychological factors to opioid misuse: A 6-month observational study. American Psychologist. 2020; 75(6): 772. <https://psycnet.apa.org/doi/10.1037/amp0000632>

***2.*** *The second major concern is about the* ***definition of chronic pain****. How do the authors define "chronic pain"? Please provide a definition : temporal definition or pathophysiological definition? It is strange that authors have considered several psychological and demographic factors to be correlated to misuse/abuse but they have not* *considered if different chronic pain syndromes could be related to different risk for misuse/abuse. Please discuss it and provide explanations (better if you can consider them)*

**Answer.** Thank you for your comment. We have used a temporal definition, according to the proposal of Treede et al., (2019). Following R#2 suggestion, the following sentence has been included in the introduction section:

“Chronic pain is pain that lasts or recurs for longer than 3 months (Treede et al., 2019)”. (line 70-71)

We found your point about *considering if different chronic pain syndromes could be related to different risk for misuse/abuse* to be very interesting. However, including more variables in this project would increase the difficulty of recruiting the sample because of having to increase the required number of participants. It is relevant to bear in mind that this project has received funding for only 4 years, and this period might not be enough if the complexity of the study is increased.

In any case, according to R#2´s suggestion, the type of chronic pain (following the IASP new classification 2019) will be included in the analysis as covariables:

“The patients’ time in pharmacological treatment with opioids, pain intensity, intake of benzodiazepines, type of chronic pain, and sex will be included as covariates in the regression analyses”. (line 377)

*3. The third concern is about the* ***sample size explanation****: the authors stated "**the sample size must be: [n = 10 * (k + 1)] (being k= number of variables). In this study we will need at least 180 participants [10 * (16+1) = 160]." How do you decide that K is 16? Please discuss it more in detail*

**Answer:** We absolutely agree with the reviewer. This is a mistake. Eighteen variables will be included in the regression model (Study 1) (see Table 2):

- Demographic, social, and medical history data:
  - Sex, age, time in pharmacological treatment with opioids, intake of benzodiazepines, and type of chronic pain (5 co-variables).
- Pain intensity
- Family and personal history of substance abuse
- History of preadolescent sexual abuse
- Specific psychological disorders (attention deficit disorder, obsessive-compulsive disorder, bipolar disorder, schizophrenia or depression).
- Anxiety and depression symptoms (2 variables)
- Post-Traumatic Stress Disorder
- Concerns about medication and medication use
- Pain catastrophizing
- Impulsiveness
- Anxiety sensitivity
- Pain acceptance
- Index of opioid misuse

Therefore, that text has changed:

“According to Freeman (49), in logistic regression analysis, the sample size must be [n = 10 * (k + 1)] (where k = number of variables). This study will require at least 190 participants [10 * (18+1) = 190]."

*4. You have stated that there will be limitations to the available data: please provide an explanation.*

**Answer:** I apologise. What we wanted to say is that as a Registered Report Protocol, we do not yet have data available to share (not even a minimal data set).

*5. It should be better to revise all the text* *with a native English speaker*

**Answer:** Following R#2’s suggestion, a professional translator (native English speaker) has reviewed the text again.
